# 1-(6-Bromo-3,4-dihydro-2*H*-1,4-benz­oxazin-4-yl)-2,2-dichloro­ethanone

**DOI:** 10.1107/S1600536812032011

**Published:** 2012-07-21

**Authors:** Fei Ye, Ying Li, Ying Fu, Li-Xia Zhao, Shuang Gao

**Affiliations:** a College of Science, Northeast Agricultural University, Harbin 150030, People’s Republic of China

## Abstract

The title compound, C_10_H_8_BrCl_2_NO_2_, is a target mol­ecule in our research on herbicide safeners. The oxazine ring has an envelope conformation, with puckering parameters close to ideal values [*Q* = 0.498 (3) Å, θ = 53.7 (3)° and ϕ = 253.4 (4)°]. The crystal structure is stabilized by C—H⋯O, C—H⋯Cl and C—H⋯Br inter­actions.

## Related literature

For general background on 1,4-benzoxazine, see: Mizar & Myrboh (2006 ▶); Macias *et al.* (2006 ▶); Tang *et al.* (2011 ▶). For the herbicide safener activity of *N*-dichloro­acetyl benzoxazine derivatives, see: Burton *et al.* (1994 ▶); Hatzios & Burgos (2004 ▶); Loniovereror (1993 ▶); Scarponi & Buono (2005 ▶). For the synthetic procedure, see: Fu *et al.* (2011 ▶).
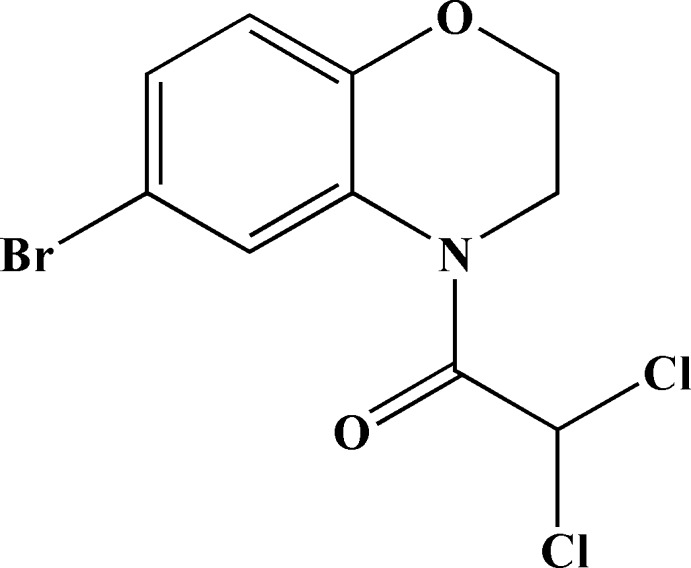


## Experimental

### 

#### Crystal data

C_10_H_8_BrCl_2_NO_2_
*M* *_r_* = 324.97
Monoclinic, 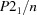

*a* = 6.8220 (8) Å
*b* = 23.567 (3) Å
*c* = 7.3746 (9) Å
β = 93.545 (1)°
*V* = 1183.4 (3) Å^3^
*Z* = 4
Mo *K*α radiation
μ = 3.91 mm^−1^
*T* = 298 K
0.40 × 0.38 × 0.28 mm

#### Data collection

Bruker SMART APEX CCD area-detector diffractometer
Absorption correction: multi-scan (*SADABS*; Sheldrick, 2002 ▶) *T*_min_ = 0.228, *T*_max_ = 0.335
11742 measured reflections
2924 independent reflections
2924 reflections with *I* > 2σ(*I*)
*R*_int_ = 0.020

#### Refinement

*R*[*F* ^2^ > 2σ(*F* ^2^)] = 0.038
*wR*(*F* ^2^) = 0.102
*S* = 1.09
2924 reflections
145 parameters
H-atom parameters constrained
Δρ_max_ = 0.44 e Å^−3^
Δρ_min_ = −0.92 e Å^−3^

### 

Data collection: *SMART* (Bruker, 1998 ▶); cell refinement: *SAINT* (Bruker, 1998 ▶); data reduction: *SAINT*; program(s) used to solve structure: *SHELXS97* (Sheldrick, 2008 ▶); program(s) used to refine structure: *SHELXL97* (Sheldrick, 2008 ▶); molecular graphics: *SHELXTL* (Sheldrick, 2008 ▶); software used to prepare material for publication: *SHELXTL*.

## Supplementary Material

Crystal structure: contains datablock(s) I, global. DOI: 10.1107/S1600536812032011/fy2057sup1.cif

Structure factors: contains datablock(s) I. DOI: 10.1107/S1600536812032011/fy2057Isup2.hkl

Supplementary material file. DOI: 10.1107/S1600536812032011/fy2057Isup3.cml

Additional supplementary materials:  crystallographic information; 3D view; checkCIF report

## Figures and Tables

**Table 1:** Hydrogen-bond geometry (Å, °)

*D*—H⋯*A*	*D*—H	H⋯*A*	*D*⋯*A*	*D*—H⋯*A*
C3—H3⋯O2^i^	0.98	2.20	3.101 (3)	153
C12—H12*B*⋯Cl2^i^	0.97	2.88	3.664 (3)	139
C11—H11*B*⋯Br1^ii^	0.97	2.99	3.853 (3)	148

Symmetry codes: (i) 
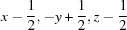
; (ii) 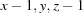.

**Fig. 1.**
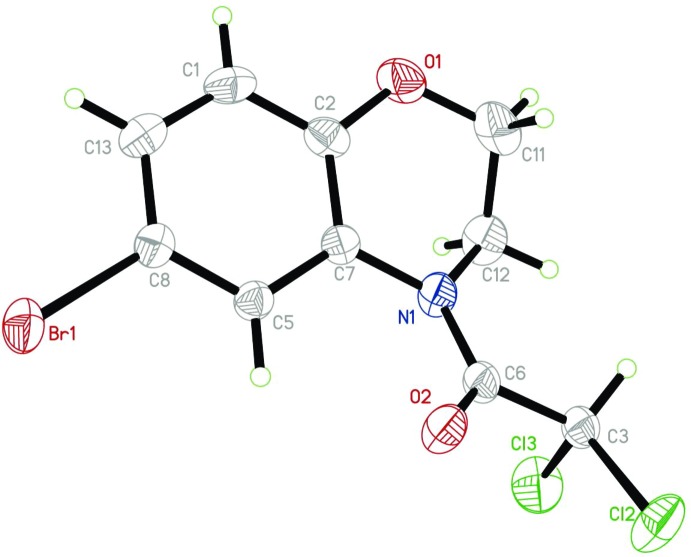
Molecular structure of the title compound with the atom labelling scheme. Displacement ellipsoids are drawn at the 30% probability level.

**Fig. 2.**
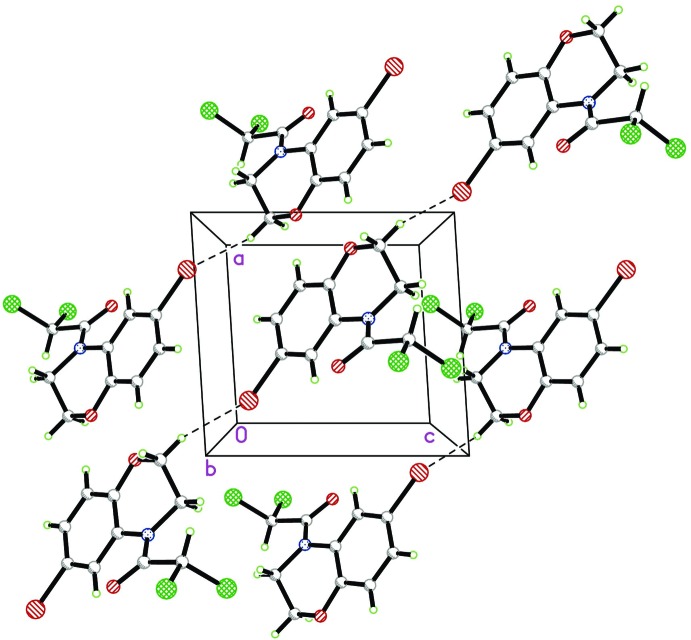
A packing diagram for the title compound, showing the intramolecular C–H···Br interaction as dashed lines.

## supplementary crystallographic information

### Comment

Substituted benzoxazine derivatives have attracted attention because of their widespread application as fungicides and insecticides (Mizar & Myrboh, 2006; Macias *et al.*, 2006; Tang *et al.*, 2011). *N*-dichloroacetyl benzoxazines have been used as herbicide safeners, which protect the crop from injury by herbicides (Burton *et al.*, 1994; Hatzios & Burgos, 2004; Loniovereror, 1993; Scarponi & Buono, 2005). As a part of our ongoing investigations of different herbicide safeners, we prepared the title compound (Fu *et al.*, 2011).

The molecular structure of the title compound is shown in Fig. 1. In the crystal, molecules are linked by weak intermolecular C—H···O, C—H···Cl, and C—H···Br interactions to form one-dimensional chains (Fig. 2, Table 1).

### Experimental

The title compound was prepared according to the literature procedure (Fu *et al.*, 2011).

The single crystal suitable for X-ray structural analysis was obtained by slow evaporation of a solution of the title compound in petroleum ether and ethyl acetate (v/v = 5:1) at room temperature.

### Refinement

All H atoms were initially located in a difference Fourier map. The C—H atoms were then constrained to an ideal geometry, with C—H distances of 0.93–0.98 Å, and with U_iso_(H) = 1.2–1.5 U_eq_(C).

### Crystal data

**Table:**

C_10_H_8_BrCl_2_NO_2_	*F*(000) = 640.0
*M**_r_* = 324.97	*D*_x_ = 1.824 Mg m^−^^3^*D*_m_ = 1.824 Mg m^−^^3^*D*_m_ measured by not measured
Monoclinic, *P*2_1_/*n*	Mo *K*α radiation, λ = 0.71073 Å
Hall symbol: -P 2yn	Cell parameters from 4705 reflections
*a* = 6.8220 (8) Å	θ = 2.8–25.8°
*b* = 23.567 (3) Å	µ = 3.91 mm^−^^1^
*c* = 7.3746 (9) Å	*T* = 298 K
β = 93.545 (1)°	Block, colourless
*V* = 1183.4 (3) Å^3^	0.40 × 0.38 × 0.28 mm
*Z* = 4	

### Data collection

**Table:**

Bruker SMART APEX CCD area-detector diffractometer	2924 independent reflections
Radiation source: fine-focus sealed tube	2924 reflections with *I* > 2σ(*I*)
Graphite monochromator	*R*_int_ = 0.020
φ and ω scans	θ_max_ = 28.3°, θ_min_ = 2.9°
Absorption correction: multi-scan (*SADABS*; Sheldrick, 2002)	*h* = −9→9
*T*_min_ = 0.228, *T*_max_ = 0.335	*k* = −31→30
11742 measured reflections	*l* = −9→9

### Refinement

**Table:**

Refinement on *F*^2^	Primary atom site location: structure-invariant direct methods
Least-squares matrix: full	Secondary atom site location: difference Fourier map
*R*[*F*^2^ > 2σ(*F*^2^)] = 0.038	Hydrogen site location: inferred from neighbouring sites
*wR*(*F*^2^) = 0.102	H-atom parameters constrained
*S* = 1.09	*w* = 1/[σ^2^(*F*_o_^2^) + (0.0487*P*)^2^ + 0.7003*P*] where *P* = (*F*_o_^2^ + 2*F*_c_^2^)/3
2924 reflections	(Δ/σ)_max_ < 0.001
145 parameters	Δρ_max_ = 0.44 e Å^−^^3^
0 restraints	Δρ_min_ = −0.92 e Å^−^^3^

### Special details

**Table:**

Geometry. All esds (except the esd in the dihedral angle between two l.s. planes) are estimated using the full covariance matrix. The cell esds are taken into account individually in the estimation of esds in distances, angles and torsion angles; correlations between esds in cell parameters are only used when they are defined by crystal symmetry. An approximate (isotropic) treatment of cell esds is used for estimating esds involving l.s. planes.
Refinement. Refinement of F^2^ against ALL reflections. The weighted R-factor wR and goodness of fit S are based on F^2^, conventional R-factors R are based on F, with F set to zero for negative F^2^. The threshold expression of F^2^ > 2sigma(F^2^) is used only for calculating R-factors(gt) etc. and is not relevant to the choice of reflections for refinement. R-factors based on F^2^ are statistically about twice as large as those based on F, and R- factors based on ALL data will be even larger.

### Fractional atomic coordinates and isotropic or equivalent isotropic displacement parameters (Å^2^)

**Table:**

	*x*	*y*	*z*	*U*_iso_*/*U*_eq_	
C11	0.5861 (5)	0.13121 (15)	0.7608 (5)	0.0693 (9)	
H11A	0.5504	0.1652	0.8251	0.083*	
H11B	0.4856	0.1243	0.6644	0.083*	
C12	0.7804 (5)	0.14020 (15)	0.6796 (4)	0.0640 (9)	
H12A	0.8180	0.1062	0.6160	0.077*	
H12B	0.7708	0.1713	0.5933	0.077*	
C13	0.9285 (5)	0.02632 (13)	1.2299 (4)	0.0535 (7)	
H13	0.9280	−0.0021	1.3173	0.064*	
C1	0.7665 (5)	0.03564 (13)	1.1156 (4)	0.0561 (7)	
H1	0.6559	0.0130	1.1240	0.067*	
C2	0.7655 (4)	0.07871 (12)	0.9869 (4)	0.0484 (6)	
C3	1.0255 (4)	0.23991 (11)	0.6714 (3)	0.0464 (6)	
H3	0.8872	0.2438	0.6289	0.056*	
O1	0.5943 (3)	0.08487 (10)	0.8818 (3)	0.0658 (6)	
C5	1.0986 (4)	0.10184 (10)	1.0846 (3)	0.0414 (5)	
H5	1.2119	0.1232	1.0742	0.050*	
C6	1.0390 (4)	0.20127 (11)	0.8395 (3)	0.0436 (6)	
C7	0.9324 (4)	0.11209 (10)	0.9693 (3)	0.0403 (5)	
C8	1.0934 (4)	0.05971 (11)	1.2139 (4)	0.0440 (6)	
O2	1.1457 (3)	0.21396 (9)	0.9706 (3)	0.0601 (6)	
N1	0.9288 (3)	0.15339 (9)	0.8282 (3)	0.0477 (5)	
Br1	1.31802 (5)	0.046715 (14)	1.37285 (4)	0.06190 (14)	
Cl2	1.12004 (16)	0.30735 (4)	0.72659 (12)	0.0759 (3)	
Cl3	1.15557 (16)	0.20811 (5)	0.49693 (13)	0.0834 (3)	

### Atomic displacement parameters (Å^2^)

**Table:**

	*U*^11^	*U*^22^	*U*^33^	*U*^12^	*U*^13^	*U*^23^
C11	0.0554 (18)	0.069 (2)	0.079 (2)	−0.0032 (15)	−0.0341 (16)	−0.0157 (17)
C12	0.072 (2)	0.0626 (18)	0.0528 (16)	−0.0159 (15)	−0.0313 (15)	−0.0025 (14)
C13	0.0639 (18)	0.0466 (14)	0.0513 (15)	−0.0081 (13)	0.0138 (13)	0.0011 (12)
C1	0.0510 (16)	0.0524 (16)	0.0665 (18)	−0.0156 (13)	0.0155 (14)	−0.0071 (13)
C2	0.0414 (13)	0.0462 (14)	0.0570 (15)	−0.0054 (11)	−0.0018 (11)	−0.0139 (12)
C3	0.0450 (13)	0.0492 (14)	0.0436 (13)	0.0012 (11)	−0.0083 (11)	0.0065 (11)
O1	0.0448 (11)	0.0669 (14)	0.0835 (15)	−0.0111 (10)	−0.0128 (10)	−0.0057 (12)
C5	0.0423 (13)	0.0382 (12)	0.0434 (12)	−0.0042 (10)	−0.0004 (10)	0.0012 (10)
C6	0.0411 (12)	0.0449 (13)	0.0430 (13)	−0.0011 (10)	−0.0121 (10)	0.0052 (10)
C7	0.0420 (13)	0.0356 (12)	0.0428 (12)	−0.0021 (10)	−0.0022 (10)	−0.0046 (10)
C8	0.0512 (14)	0.0406 (13)	0.0405 (12)	0.0024 (11)	0.0043 (11)	0.0002 (10)
O2	0.0667 (13)	0.0535 (11)	0.0559 (11)	−0.0221 (10)	−0.0302 (10)	0.0168 (9)
N1	0.0520 (13)	0.0427 (11)	0.0455 (11)	−0.0083 (10)	−0.0194 (10)	0.0016 (9)
Br1	0.0668 (2)	0.0634 (2)	0.05402 (19)	0.00348 (14)	−0.00809 (14)	0.01693 (13)
Cl2	0.1089 (7)	0.0562 (5)	0.0599 (5)	−0.0264 (4)	−0.0170 (4)	0.0171 (4)
Cl3	0.0936 (7)	0.0934 (7)	0.0651 (5)	0.0173 (5)	0.0213 (5)	0.0018 (5)

### Geometric parameters (Å, º)

**Table:**

C11—O1	1.409 (4)	C2—C7	1.397 (4)
C11—C12	1.503 (5)	C3—C6	1.536 (3)
C11—H11A	0.9700	C3—Cl2	1.754 (3)
C11—H11B	0.9700	C3—Cl3	1.774 (3)
C12—N1	1.479 (3)	C3—H3	0.9800
C12—H12A	0.9700	C5—C8	1.379 (4)
C12—H12B	0.9700	C5—C7	1.396 (3)
C13—C1	1.365 (5)	C5—H5	0.9300
C13—C8	1.384 (4)	C6—O2	1.211 (3)
C13—H13	0.9300	C6—N1	1.356 (3)
C1—C2	1.390 (4)	C7—N1	1.424 (3)
C1—H1	0.9300	C8—Br1	1.895 (3)
C2—O1	1.369 (3)		
			
O1—C11—C12	111.1 (3)	C6—C3—Cl3	109.09 (19)
O1—C11—H11A	109.4	Cl2—C3—Cl3	110.98 (16)
C12—C11—H11A	109.4	C6—C3—H3	108.8
O1—C11—H11B	109.4	Cl2—C3—H3	108.8
C12—C11—H11B	109.4	Cl3—C3—H3	108.8
H11A—C11—H11B	108.0	C2—O1—C11	116.1 (2)
N1—C12—C11	108.3 (3)	C8—C5—C7	119.5 (2)
N1—C12—H12A	110.0	C8—C5—H5	120.3
C11—C12—H12A	110.0	C7—C5—H5	120.3
N1—C12—H12B	110.0	O2—C6—N1	123.9 (2)
C11—C12—H12B	110.0	O2—C6—C3	120.1 (2)
H12A—C12—H12B	108.4	N1—C6—C3	116.0 (2)
C1—C13—C8	119.2 (3)	C5—C7—C2	118.8 (2)
C1—C13—H13	120.4	C5—C7—N1	122.7 (2)
C8—C13—H13	120.4	C2—C7—N1	118.4 (2)
C13—C1—C2	120.6 (3)	C5—C8—C13	121.6 (3)
C13—C1—H1	119.7	C5—C8—Br1	119.3 (2)
C2—C1—H1	119.7	C13—C8—Br1	119.1 (2)
O1—C2—C1	115.6 (3)	C6—N1—C7	122.7 (2)
O1—C2—C7	124.0 (3)	C6—N1—C12	124.9 (2)
C1—C2—C7	120.4 (3)	C7—N1—C12	112.2 (2)
C6—C3—Cl2	110.32 (17)		

### Hydrogen-bond geometry (Å, º)

**Table:**

*D*—H···*A*	*D*—H	H···*A*	*D*···*A*	*D*—H···*A*
C3—H3···O2^i^	0.98	2.20	3.101 (3)	153
C12—H12*B*···Cl2^i^	0.97	2.88	3.664 (3)	139
C11—H11*B*···Br1^ii^	0.97	2.99	3.853 (3)	148

Symmetry codes: (i) *x*−1/2, −*y*+1/2, *z*−1/2; (ii) *x*−1, *y*, *z*−1.
